# A novel maturity index for assessing medical device startups

**DOI:** 10.1017/cts.2022.436

**Published:** 2022-08-01

**Authors:** Frances J. Richmond, Grzegorz Zapotoczny, Brian Green, Sowmya Lokappa, Katy Rudnick, Juan Espinoza, the ICRS-CTIP Collaborative for Regulatory Research

**Affiliations:** 1 Department of Regulatory and Quality Sciences, School of Pharmacy, University of Southern California, Los Angeles, CA, USA; 2 Department of Pediatrics, Children’s Hospital Los Angeles, Los Angeles, CA, USA; 3 Department of Pediatrics, Keck School of Medicine, University of Southern California, Los Angeles, CA, USA

**Keywords:** Pediatrics, medical devices, regulatory science, business maturity, startups, clinical readiness, small- and medium-sized enterprises

## Abstract

**Background:**

Startup companies in the healthcare sector often fail because they lack sufficient entrepreneurial, regulatory, and business development expertise. Maturity models provide useful frameworks to assess the state of business elements more systematically than heuristic assessments. However, previous models were developed primarily to characterize the business state of larger nonmedical companies. A maturity index designed specifically for startup companies in the medical product sector could help to identify areas in which targeted interventions could assist business development.

**Methods:**

A novel MedTech Startup Maturity Index (SMI) was developed by a collaborative team of academic and industry experts and refined through feedback from external stakeholders. Pediatric medical device startups associated with the West Coast Consortium for Technology & Innovation in Pediatrics (CTIP) were scored and ranked according to the SMI following semi-structured interviews. The CTIP executive team independently ranked the maturity of each company based on their extensive experiences with the same companies.

**Results:**

SMI scores for 16 companies ranged from 1.2 to 3.8 out of 4. These scores were well aligned with heuristic CTIP rankings for 14 out of 16 companies, reflected by strong correlations between the two datasets (Spearman’s rho = 0.721, *P* = 0.002, and Kendall’s tau-b = 0.526, *P* = 0.006).

**Conclusions:**

The SMI yields maturity scores that correlate well with expert rankings but can be assessed without prior company knowledge and can identify specific areas of concern more systematically. Further research is required to generalize and validate the SMI as a pre-/post-evaluation tool.

## Introduction

Modern healthcare depends on a constant infusion of novel medical products, often developed in startup companies [1,2]. The increasing importance of their contributions is reflected by the growth of licensing activity at universities [3] and of medically related Small Business Innovation Research (SBIR) and Small Business Technology Transfer (STTR) funding over the last decade [4]. Several accelerator and incubator programs have been established at universities and academic medical centers to support and nurture these early-stage companies, often with a combination of federal and institutional funds [5,6]. However, translation of biomedical technology is challenging because its developmental path is shaped not only by the typical growth and product development problems of small companies in general [7] but also by regulatory, clinical, and business hurdles. The many years of laboratory, animal, and clinical testing [8] have business risks and make development both costly and slow. Most startup companies do not have sufficient funding to cross the “valley of death,” the period before substantial investment in development can be recovered from product sales [9–11]. To persist in the market, the companies must attract the interest of investors, by showing not only that the product/technology addresses an unmet medical need but also that the business has intellectual property protection, a solid management plan, a sizable market niche, and substantial revenue promise [12].

Government agencies have been concerned about the problems faced by smaller businesses and have developed policies and support systems for them. These programs include targeted funding through the SBIR/STTR programs that share a budget of over $3.7 billion/year [13,14]. The US Food and Drug Administration (FDA) also has programs aimed to foster the scientific and regulatory capacity of smaller medical product companies [15]. These include targeted educational and support programs such as FDA’s Pediatric Device Consortia that provide grants and consulting services [16]. Non-profit groups may also approach “angel” investors or venture capitalists for early-stage funding. Those early-stage investors, in turn, must decide on a portfolio of small companies most likely to be successful commercially and provide them with the targeted funding, management support, and training to accelerate progression toward their business goals.

However, it is challenging for investors to understand companies in their portfolio well enough to design interventions that the small businesses need to develop most efficiently. Many early-stage entrepreneurs in the MedTech field lack business and regulatory experience and may not be able to self-diagnose their company’s deficiencies. Investors then must rely on time-consuming heuristic approaches based on company presentations, documentary review, or personal interactions to assess the companies. Those investors anecdotally identify the need for more systematic profiling tools. In other sectors, companies have been evaluated and compared using maturity tools such as the popular Capability Maturity Model (CMM) [17]. As described by Mettler and colleagues [18], these tools define maturity as “an evolutionary progress in the demonstration of a specific ability or in the accomplishment of a target from an initial to a desired or normally occurring end stage. The purpose of maturity models is to give guidance through this evolutionary process by incorporating formality into the improvement activities” [18]. The CMM and related models have since been applied widely as tools to stage a diversity of process and software development programs [17,19]. More recently, maturity models have also been developed to evaluate businesses more broadly [20]. However, the models are usually designed for medium to large enterprises and can be relatively high level in scope or focused on certain types of businesses. They are often not suitable to assess the rather different and specific needs of startup companies [7]. In a recent comprehensive review of business maturity models, Virkkala and colleagues [21] pointed out that only two of the many models identified in their study even mentioned small- and medium-sized enterprises; they advocated for the development of “micro-enterprise-focused maturity models.” Further, the few currently available maturity tools that do exist are typically directed at small companies specializing in, for example, tourism or manufacturing operations that do not experience the types of impediments associated with medical products and may not have significant growth as an objective [22,23].

To our knowledge, no specifically designed model has been developed to assess the business maturity of medical product startups. The models or frameworks in the medical product field that do exist such as the Stanford model of BioDesign and the FDA’s Total Product Life Cycle [24–26] are typically directed at describing the evolution of the product under development. However, medical product startups also struggle from a business perspective as they deal with the stresses imposed by the longer development timelines and complex regulatory and reimbursement requirements of their product. A tool to assess the business maturity of medical device startups across several domains would serve many purposes. It could assist companies in determining the startup’s current state and best path forward, help educators, accelerators, and government agencies to verify that their support programs are meeting industry needs and allow investors to profile and compare a portfolio of companies. Here we describe and validate a Startup Maturity Index (SMI) that pays homage to the well-known CMM and its many variants used by academicians, consultants, and governments [17,23,27,28]. All of these models divide the evolutionary path into a number of graduated levels. The seminal CMM index, [19] for example, identified five levels, designated as *initial, repeatable, defined, managed, and optimizing*. These models also identify a series of “swim lanes” – process domains that can be assessed by examining the state of certain types of activities that might characteristic of each level of maturity. The modified SMI was then used to characterize the strengths and weaknesses of a cohort of pediatric medical device startups. A scoring system was applied, not for the specific purpose of grading the companies but rather to facilitate and early-stage validation of the approach by comparing the scores to comparable arm-length ratings assigned independently by experienced support teams working with those companies.

## Methods

### Instrument Development

A survey instrument was developed by a multistakeholder working group, the USC ICRS Research Collaborative, composed of 19 academic, regulatory, and industry experts experienced in medical product development. Maturity levels were modified from those of Paulk and colleagues (1993) [19] to remove the final stage of “optimizing,” found in models for more established companies, because the Collaborative believed that it is unlikely that startups would reasonably reach such a stage. The levels were defined as Initial (1), Foundational (2), Managed (3), and Mature (4), because the Collaborative believed these to be descriptors that would be understood better by the ultimate users in companies and support agencies but recognized that any such descriptors would be arbitrary. Based on existing literature, prior maturity models, and group discussion, the Collaborative selected five domains related to different aspects of business process: a) Business Vision, b) Business Logistics, c) Design and Production Capabilities, d) Regulatory and Clinical Readiness, and e) Human Resources and Role Evolution. The Collaborative then developed 6–10 readiness questions that were used to probe the state of development of certain subordinate elements believed to be associated with each process domain.

To identify companies with which to validate the tool, The ICRS Research Collaborative partnered with the West Coast Consortium for Technology & Innovation in Pediatrics (CTIP), an FDA-funded, pediatric medical device accelerator centered at Children’s Hospital Los Angeles (CHLA) and the University of Southern California (USC). The eleven member institutions of CTIP support and counsel over 100 early-stage pediatric medical device companies distributed across the country. Eight companies from the CTIP portfolio volunteered to participate in a first round of 30–40 min, semi-structured interviews carried out using the videoconferencing tool, Zoom. Each interview was conducted with senior executives of the participating companies by a team of 2–4 interviewers from the Collaborative. The interviewee was invited to briefly summarize the mission and history of the company under study, as well as their future plans, including exit strategy, and then asked to respond to each of the SMI questions presented in sequence. The criteria used to score the maturity levels were not shared with the interviewees so that the interviewees would not frame their responses by attempting to self-score into a higher category. To address inter-rater consistency, individual members of the interviewing team independently scored the maturity level suggested by the answers in each domain. Immediately after the interview, team members discussed the score assigned for each domain in sequence. In a very few instances where team members had different scores, the discussion continued until consensus was reached. The scores were collated and used to calculate an overall maturity score for each of the domains and for the company as a whole, by averaging the summed scores of the 5 domains. If a question was not applicable to a company’s unique business case (e.g., companies developing software might not have manufacturing facilities), the question was marked as not applicable and was excluded from the scoring assessment, with appropriate adjustment of the denominator.

Four members of the development team reviewed the materials from the first round of eight interviews. Based on feedback from the initial interviewers and the Consortium, the development team implemented survey revisions, a step that removed two questions as described below, and modified the wording of others to improve clarity and relevance. The refined SMI (Appendix 1 full instrument) was reviewed by other developers from the Collaborative. A second cohort of eight companies was then interviewed and scored in the same manner as the first cohort. Once all 16 companies were scored, they were ranked from 1 to 16 based on their SMI score, with 1 being the most mature. When two companies shared equal scores, they were given the same ranking.

### Instrument Validation

As a comparator to validate the survey, the CTIP team scored the overall maturity of the same 16 companies on a scale from 1 to 4 (with 4 as most mature) based on their previous interactions and past assessments of those same companies. The CTIP team did not use the SMI survey and did not receive directive instructions to shape their maturity assessments. All participating companies had been in the CTIP portfolio for at least 12 months at the time of the exercise, so the CTIP team maturity assessment was considered to provide a heuristic “ground truth.” In addition to assigning a maturity score, the CTIP team also ranked all companies in terms of maturity from 1 to 16, with 1 being considered the most mature.

The scores and rankings of the SMI were compared to those of CTIP using a three-step process. First, the alignment between the two independent maturity assessments was calculated using formula:

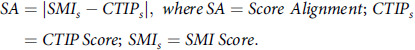




The alignment of company scores was considered “high” when SA ≤ 0.6, “medium” between 0.7–1.2, and “low” when ≥1.3. Second, the alignment between the two independent rankings of each company was assessed using the formula:

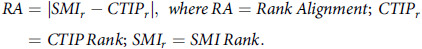




The alignment of company rankings was considered “high” when RA ≤ 3, “medium” between 4–6, and “low” when ≥7. Finally, Overall Alignment was derived by compiling the two sets of descriptors, “low,” “medium,” or “high,” into a single value. If there was concurrence between *Score Alignment* and *Rank Alignment*, then the *Overall Alignment* was considered “low,” “medium,” or “high” accordingly; however, if the *Score Alignment* and *Rank Alignment* differed from each other, the *Overall Alignment* was a concatenation of the two: “low-medium” or “medium-high.”

### Statistical Analysis

Two methods were used to examine the correspondence between rankings. The strength of the correlation between the ranks of two rating systems was estimated by Spearman’s correlation coefficient [29,30]. It is based on Pearson’s correlation computed on the ranks and average ranks. Kendall’s Tau was calculated as the ratio of the difference between concordant and discordant parts and the total number of possible pairs of ranks of two raters [31,32]. It has been extended to account for possible ties of ranks [33]. H_o_ was defined as rankings being independent. Kendall’s Tau was corrected for ties and continuity. STATA 17.0 (College Station, TX) was used to compute and test these measures of association.

## Results

### Participating Companies

A total of 16 startups participated across two rounds of interviews. Table 1 provides an overview of key descriptions of their device, development, regulatory considerations, and fundraising status. The names of companies are anonymized; they are ordered in a manner that does not reflect their scores. Nearly half of these startups (7 of 16) had fewer than three team members while two startups had ten or more team members. There was no apparent correlation between team size and maturity.

**Table 1.** Characteristics of companies participating in the evaluation and validation of the Startup Maturity Index

Clinical focus	Device stage	Anticipated device class	Anticipated regulatory pathway	Number of team members	Fundraising stage
Orthopedics	Concept	Class I	510(k) exempt	Less than 3	n/a
General hospital devices	Manufacturing	Class I	510(k) exempt	Less than 3	Seed
Orthopedics	Commercial use	Class I	510(k) exempt	3 to 10	Seed
Ophthalmology	Marketing	Class I	510(k) exempt	Less than 3	Seed
Obstetrics and gynecology	Prototype	Class I/Class II	510(k) exempt	Less than 3	Grants only
Neonatology	Prototype	Class II	510(k)	3 to 10	Grants only
Urology	Concept	Class II	510(k)	Less than 3	Grants only
Emergency medicine	Advanced prototype	Class II	510(k)	3 to 10	Seed
Radiology	Advanced prototype	Class II	510(k)	3 to 10	Grants only
Microbiology diagnostics	Advanced prototype	Class II	510(k)	Less than 3	Seed
Neurology	Clinical testing	Class II	510(k)	3 to 10	Seed
Mental health	Clinical testing	Class II	510(k)	More than 10	Series A
Infection control	Concept	Class II	510(k)	Less than 3	n/a
General hospital devices	Manufacturing	Class II	510(k)	More than 10	Series B
Physical rehabilitation	Commercial use	Class II	510(k) exempt	3 to 10	Seed
Allergy & immunology	Advanced prototype	Combination product	TBD	3 to 10	Seed

n/a = Not applicable; TBD = to be determined.

### Maturity Scores and Ranking

SMI and CTIP maturity scores and rankings are shown in Table 2. The alignments between SMI and CTIP assessments were at least medium-high for 14 of the 16 companies. The alignment for the other two companies was designated as medium (Company F) and low-medium (Company L), respectively. Statistically, the SMI and CTIP rankings were highly correlated: Spearman’s rho = 0.721, *P* = 0.002, and Kendall’s tau-b = 0.526, *P* = 0.006.

**Table 2.** Variance and alignment of scores and rankings

CTIP rank	SMI rank	Company	SMI score	CTIP score	Overall alignment
15	16	**A**	1.2	1	High
13	14	**B**	1.9	1	Medium-High
14	14	**C**	1.9	1	Medium-High
7	13	**D**	2	2	Medium-High
9	12	**E**	2.1	2	High
16	11	**F**	2.2	1	Medium
10	9	**G**	2.8	2	Medium-High
5	9	**H**	2.8	3	Medium-High
11	8	**I**	2.9	2	Medium-High
4	6	**J**	3.1	3	High
8	6	**K**	3.1	2	Medium-High
12	5	**L**	3.2	2	Low-Medium
1	3	**M**	3.3	4	Medium-High
2	3	**N**	3.3	4	Medium-High
6	2	**P**	3.7	3	Medium-High
3	1	**Q**	3.8	4	High

SMI = Startup Maturity Index, CTIP = West Coast Consortium for Technology & Innovation in Pediatrics.

Table 3 shows the SMI questions organized by domain and the average score across all 16 companies. In the Business Vision domain, companies were typically better at articulating their purpose, leadership, and target indication(s), but scored lower on profiling the target product and customer base. In the Business Logistics domain, most companies had relatively high scores on protecting intellectual property and assuring financial support but were typically weaker in planning for growth and understanding core business practices, such as securing permits and licenses, instituting accounting practices, and adopting governance policies.

**Table 3.** Startup Maturity Index questions and score from 16 companies

Questions	Average score	SD
*Business Vision*	*3.0*	*0.69*
1. Have you defined your business purpose and mission/vision statement?	2.8	1.23
2. Have key milestones been identified?	3.2	0.85
3. Has a leadership team been established?	3.2	0.70
4. Do you have a Target Product Profile (TPP) outlining desired product characteristics?	3.0	1.32
5. Have disease prevalence and/or market positioning been identified?	3.4	0.63
6. Have you surveyed the defined customer population?	3.0	0.83
7. Have you performed a SWOT analysis for your company?	2.8	1.00
*Business Logistics*	*2.8*	*0.82*
8. What intellectual property protection do you have, if applicable?	3.4	0.89
9. What types and amounts of funding do you have?	2.6	0.89
10. Have you determined the structure of your business entity, for example, LLC, INC, S-corp?	3.0	0.97
11. Do you have a physical location and if so, have you determined the required permits and licenses?	2.0	0.88
12. Are you aware of accounting needs and practices?	2.8	0.88
13. What is the governance structure of your business?	2.8	1.25
*Design and Production Capabilities*	*2.6*	*1.06*
14. Do your facility/utility/storage requirements & capabilities match your office & manufacturing needs?	2.3	1.07
15. How is your equipment for manufacturing and testing validated and maintained?	2.7	1.49
16a. Do you understand and document processes for producing the product?	2.5	1.29
16b. Do you understand and document processes for developing and validating the software?	3.2	0.84
17. What are the environmental, health, and safety considerations for your production facility?	2.7	1.60
18. Do you control the specifications and sourcing of major raw materials/ components/ supplies?	2.8	1.03
19. What is the state of your IT & network infrastructure?	2.2	1.17
*Regulatory and Clinical Readiness*	*2.6*	*0.72*
20. Do you understand how to develop and register medical devices in accordance w/US medical device regulations?	3.0	0.87
21. Do you have a QMS to formally document processes and procedures to attain high reproducibility?	2.6	0.99
22. Do you have staff to develop an insurance reimbursement strategy?	2.5	1.08
23. Are you familiar with permissible promotion and sales practices for medical devices?	1.8	0.89
24. Has a clinical focus group evaluated the medical need and design concept for your device?	2.8	1.02
25. Do you have clinical assistance or formal partnerships in the development of the device?	2.9	1.06
Do you have access to clinical partnerships? *(round 1 only)*	2.6	1.19
*Human Resources and Role Evolution*	*2.4*	*1.07*
26. Do you have Human Relations (HR) structures and policies?	1.7	1.07
27. Have you defined the roles, titles, and responsibilities of the staff?	2.8	0.90
28. Are you compliant with federal, state, and local labor laws?	2.6	1.26
29. Do you have a system to ensure that staff are paid on time and appropriate taxes are withheld?	2.7	1.23
30. Do you keep employee records, and if so, how do you keep this information confidential?	2.2	1.20
How do you recruit, hire, and fire employees? *(round 1 only)*	1.6	1.13
**SMI Overall Score**	**2.7**	**0.72**

SD = Standard deviation; SWOT = Strengths, Weaknesses, Opportunities, Threats; IT = Information Technology; QMS = Quality Management System.

**Fig. 1.**
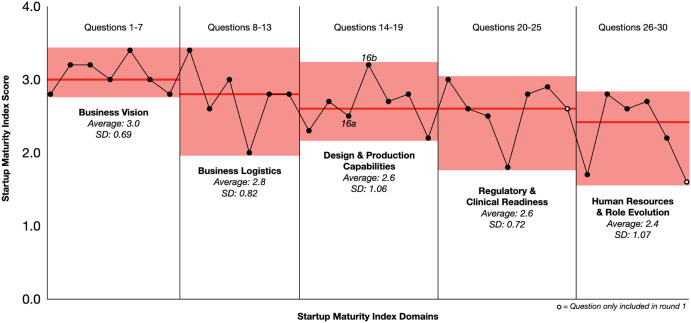
Graphical summary of Startup Maturity Index (SMI) Scores across five domains. The x-axis indicates SMI questions 1–30 and y-axis the corresponding SMI scores. The red horizontal bars represent the section average score, while the red highlighted area represents the range of scores. Each individual point represents the average score across all companies for a given question, sequentially from left to right. The two points with a white center represent questions that were only included in the first round of interviews. SD = Standard deviation.

In the Design and Production domain, the least mature companies had not yet initiated production activities, such as software validation or IT refinement. Notably, 4 of the 16 companies were developing Software as a Medical Device, rendering a few questions relating to manufacturing, equipment, raw materials, or environmental health as “not applicable.” Also, several companies relied on third-party manufacturers. Although the companies are required to oversee these outsourced activities, several participants stated that they rely on the outsourcing firm for production documents such as standard operating procedures, quality certifications, and production records. In the Regulatory/Clinical domain, most companies had used clinical focus groups or clinical partnerships to evaluate the medical need and design concept for the device and, in some instances, to plan clinical trials. However, most scored lower in their understanding of permissible promotion and sales practices that come into play even before a medical product is commercialized. Figure 1 is a graphical representation of Table 3, showing the score averages and ranges for each question and domain for all 16 companies.

**Fig. 2.**
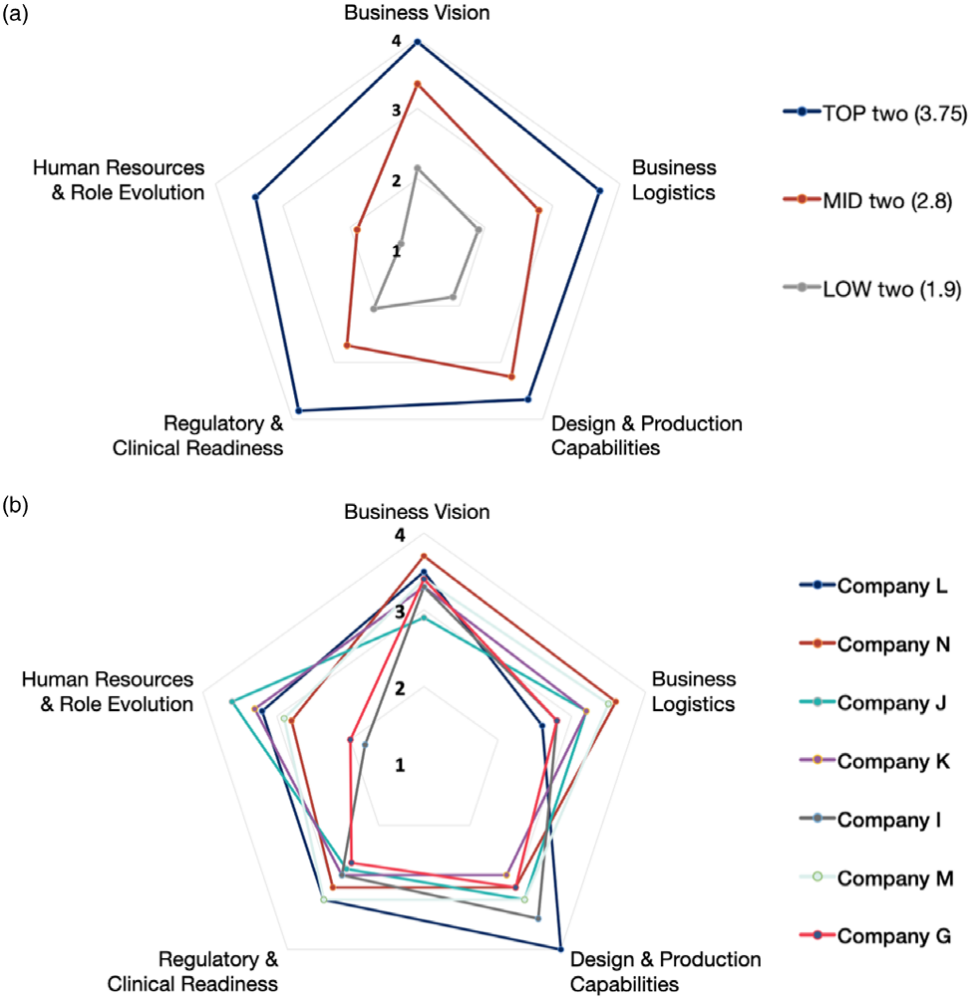
Polar plots representing Startup Maturity Index (SMI) scores for selected companies. Figure 2a: SMI scores across 5 assessed domains in the companies with 2 highest SMI scores (averaged and shown in blue); 2 medium SMI scores (averaged and shown in orange); 2 lowest SMI scores (averaged and shown in gray). Figure 2b: SMI scores across 5 assessed domains in companies with highest variations between the domain scores.

The extent of variation in scores between companies across domains can be illustrated by the polar plots in Fig. 2. Overall scores were broadly consistent with scores on each of the domains, particularly for the most mature companies (Fig. 2A). Companies with lower SMI scores typically had weaker scores in the Human Resources and Role Evolution domain compared to the other domains. The domain-related variation was also wider for less mature companies, shown for 7 representative companies with overall SMI scores between 2.8 and 3.3 (Fig. 2B). The highest variability in scores across companies was in the Human Resources and Role Evolution domain. Many companies had a poor understanding of legal, business, and payment practices related to human resources. This immaturity was often attributed to the fact that the company had few employees or outsourced human resource management to a specialized management firm, and thus, many questions were considered “not applicable.”

## Discussion

A tool such as the SMI to measure maturity offers several benefits to systematize the evaluation of business capabilities. It provides investors and accelerators the means to compare the current state and progress of a range of companies in its early-stage portfolio. It helps academic support groups and accelerators to circumvent the much longer and more easily biased assessments made based on the nature and collegiality of personal interactions, gender, and race [34–37]. By using a systematized and methodical rating scale for different domains, the SMI helps to highlight areas in which interventions could be planned more strategically and limited resources could be directed more wisely. However, the tool must match the needs and stages of the type of business that it serves. Thus, the structure of the SMI tool was modified to have four rather than five levels; the fifth level common in previous models assumes the presence of business features well beyond what can be reasonably expected for startups. The descriptors of these levels were also modified to reflect stages more appropriate for small businesses. Finally, the SMI addressed domains of considerable importance to medical product companies, such as regulatory, clinical, and reimbursement concerns, that are less relevant to companies outside of the medical products sector.

A key requirement when developing maturity tools is to design an appropriate method for their validation. Here, the maturity assessments of the SMI were compared to the heuristic evaluations made by CTIP program leaders knowledgeable about the studied companies. This validation paradigm was made possible because we could recruit a relatively large case series of small companies all focused on [10,38]. These companies had been well-characterized with significant amounts of data already available in the CTIP database and with regular access to the CTIP executive team. Each evaluator on the CTIP team had years of experience working with medical device startups across the major domains considered by the Collaborative to be important and so were able to provide comprehensive evaluations of each company. This degree of familiarity is unusual. The process of due diligence and evaluation of a startup can be highly subjective and imprecise, particularly among early-stage companies where many of the typical metrics used to assess the capabilities of more mature companies do not yet exist. The CTIP organization arranges frequent interactions between SMEs and a well-qualified advisory team of experts and therefore has good insight into the strengths and weaknesses of each company. However, many academic accelerators and incubators do not have the medical, engineering, regulatory, and reimbursement expertise to perform such a broadly based evaluation.

The design and structure of the SMI lends itself not only to evaluating companies but also to giving them a feedback and education tool. In this study, the SMI was administered by an assessment team, but in practice it could be completed by companies on their own or with minimal support (though anecdotally, most of the respondents preferred the interactive interview format). Through the domains and questions, the participant is presented with a framework to illustrate what a company might do to progress toward a more advanced maturity stage. By comparing the company’s capabilities to this framework, the participants can identify strengths, weaknesses, and potential areas where attention could be beneficial. This can inform a “roadmap” to guide their further development, in a manner like that used for Operative Performance Rating Scales [39–41] designed to improve the skills of healthcare professionals, or Informatics Maturity Models, which serve as real-time feedback and teaching tools [42]. The second group of eight companies was asked for feedback about the tool and its usefulness; all were broadly positive about its educational and strategic value.

The SMI could be leveraged by academic accelerators and incubators to formalize and guide feedback to companies in their portfolios or to select projects matched most closely to their capabilities and resources. Investors could also benefit from using the SMI to systematically evaluate potential investments. Our findings suggest that the SMI could highlight areas in which intervention might be most helpful. For example, it is often assumed that intellectual property and regulatory requirements are the greatest hurdles for small companies, but these results suggest that many of the studied companies have significant educational needs related to documentation management, accounting methods, and human resource planning.

The strong alignment between the two sets of evaluations conducted here gives confidence that the SMI accomplishes its objectives. Although CTIP is a MedTech accelerator dedicated to supporting companies developing products with pediatric indications, only 1 out of 16 did not also have a potential adult application. We believe the SMI questions are relevant to other types of medical device companies. What this study does not do, however, is assure that the results seen for the companies in this case series are typical for medical device startups elsewhere. Early-stage companies in the CTIP portfolio, with its particular focus on pediatrics, have better access to consulting and educational resources than most, so their maturities in certain domains may exceed the maturity of early-stage startups more generally. In particular, many MedTech startups struggle with regulatory and clinical readiness [43,44], underlining the importance of specialized accelerators like the Pediatric Device Consortia program [16]. Further, none of the companies surveyed were developing Class III devices that would enter the market through the premarket approval route. Bringing a class III device to market can cost up to $100 million and take 7 to 10 years [45]. The SMI assumes that the companies have reached the point of having a team, so can produce misaligned results when working with solo founders, as it did here with company L. Further validation is also needed to understand if this tool would be useful for digital health companies, pharmaceutical companies, or companies making other types of healthcare products.

Our goal for this study was to develop a tool for the rapid assessment of business maturity for medical device startup companies. Moving forward, we intend to collect additional data and disseminate the tool to gain feedback from other accelerators and incubators. We hope that other institutions and MedTech accelerators will consider using this or a modified version of the SMI and share their experiences in follow-up publications. We continue to refine and evaluate the SMI in a variety of contexts, such as working with federal agencies like NIH, SBA, and FDA to improve their evaluation processes of startups. Appendix 1 contains the version of the SMI that was used for this study; please contact the authors for the latest version.

## Conclusion

The SMI produces maturity scores and rankings for early-stage MedTech startups that correlate highly with expert insights. This tool could be used by institutions to select startups for their accelerators and target specific areas of support. Further research is required to assess its validity in other settings and sectors, and its potential as a pre-/post-evaluation tool. We hope that other organizations find this tool useful as they work to support medical device innovation.

## Supplementary material

For supplementary material accompanying this paper visit https://doi.org/10.1017/cts.2022.436. click here to view supplementary material
