# Supplementary material for: A novel maturity index for assessing medical device startups
Source: J Clin Transl Sci. 2022 Aug 1;6(1):e99. doi: 10.1017/cts.2022.436 (PMC9428664; doi:10.1017/cts.2022.436)
Supplement: Supplementary file 1 [file S2059866122004368sup001.docx]

**STARTUP MATURITY INDEX**

**Introduction**

**The Startup Maturity Index encompasses potential areas of generalized development and is not intended to be a stepwise formula for business development, nor is it a checklist for project success. Many of the listed items for each development area may not be applicable and can be noted as such in the ‘observations’ column with no penalty to the overall maturity determination. Further, please request clarification if a topic is not fully understood as there are scenarios which may fall outside the index. Some areas of development may be contracted or virtual and these can also be noted in the observations.**

**Before the interview commences, please provide a brief overview of your company and product.**

**Table 1: Business Vision**

| **Development Topic** | **Question** | **Maturity Gradient Scale** | | | | **Observations** |
| --- | --- | --- | --- | --- | --- | --- |
|  |  | **Initial** | **Foundational** | **Managed** | **Mature** |  |
| **Business Intent:**  **Vision/Desire/**  **Commitment** | **1.Have you defined your business purpose and mission/vision statement?** | We have not developed our business purpose or mission/vision statement. | We have drafted our business purpose and mission/vision statement. | We have created our business purpose and vision/ mission statement, focused on company objectives as part of our business plan. | We have a business purpose /vision clearly defined and linked to long-term strategy. |  |
|  | **2.Have key milestones been identified?  (checkpoints for success, go/no go decision points)** | We have not identified milestones for company success. | We have proposed milestones for success though they are still very broad. | We have specific milestones for success, but priorities & decision points are not clearly defined. | We have key milestones and deliverables with defined pathways and monitoring to ensure stakeholder communications and decision checkpoints. |  |
|  | **3.Has a leadership team been established?** | We have not established a leadership team. | We proposed a leadership team, but we have not specified their key functions. | We have defined the leadership team but priorities and commitment to the company remain unclear. | We have an experienced leadership team committed to the company’s vision. |  |
| **Competitive Landscape:**  **Unmet need/ Differentiation** | **4.Do you have a Target Product Profile (TPP) outlining desired product characteristics: intended use, target populations, safety, and efficacy?** | We are not familiar with the content of a TPP. | We are developing a TPP focusing on target indications and claims. | We created a draft TPP and are beginning to define the potential economic value of the product. | We have a TPP with a plan for differentiation/ exclusivity and a mechanism for updating as intelligence is gathered. |  |
|  | **5.Have disease prevalence and/or market positioning been identified?** | We are not aware of the disease prevalence or market needs. | We have conducted research on disease prevalence and the market needs. | We can identify our direct competitors and their market share but not their specific strengths/ weaknesses. | We have identified key features of two or more largest competitors and used them for product planning purposes. We have a procedure for monitoring competitors. |  |
|  | **6.Have you surveyed the defined customer population?** | We have not identified our customer population. | We have identified our customer population but have not surveyed them. | We have identified and surveyed our customer population. | We have created focus groups to optimize the product based on the survey of our customer population. |  |
| **SWOT** | **7.Have you performed an analysis of strengths, weaknesses, opportunities, and threats (SWOT analysis) for your company?** | We have not performed the SWOT analysis. | Our SWOT is based on gut feel with only a few strengths and opportunities listed. | Our SWOT is beginning to incorporate research to define strengths, opportunities, weaknesses, and threats related to commercialization. | We developed a comprehensive SWOT with a contingency plan to alleviate weaknesses, counteract threats, and a monitoring plan to adjust as conditions change. |  |

**Table 2: Business Logistics**

| **Development Topic** | **Question** | **Maturity Gradient Scale** | | | | **Observations** |
| --- | --- | --- | --- | --- | --- | --- |
|  |  | **Initial** | **Foundational** | **Managed** | **Mature** |  |
| **Legal – Intellectual property protection** | **8.What intellectual property protection do you have, if applicable?** | We have no intellectual property protection and have not explored the patent landscape. | We have IP in review; we understand the IP landscape but have not explored it systematically. | We have a beginning patent/trademark position and have a cursory exploration of the patent landscape. | We have strong IP property protection and have secured qualified legal services. |  |
| **Financial – Funding** | **9.What types and amounts of funding do you have?** | We are funded by personal assets sufficient for one-year needs. | We are seeking funds from small business govt programs and grants from private organizations. We have participated in a few pitch sessions. | We have secured first-round venture capital with funding sufficient to advance development into later stages. | We have secured later-round venture capital sufficient to fund late-stage development and market entry. We have explored partnerships with other companies to advance business objectives. |  |
| **Structure of business entity** | **10.Have you determined the structure of your business entity e.g., LLC, INC, S-corp?** | We have not yet sought a defined business structure or designation. | Our company is structured as a single proprietorship or partnership. | Our company is structured as a corporation. | Our well-structured business identity is supported by accounting and legal counsel with oversight from an arm’s length advisory board. |  |
| **Licenses, Permits, and Registration of the Business** | **11.Do you have a physical location and if so, have you determined the required permits and licenses?** | We have no dedicated physical location or office/laboratory space for our company. | Our dedicated physical location includes office ad laboratory space sufficient for immediate/ short-term needs | We have a footprint of office and laboratory space adequate for development activities and initial milestones; applicable permits and licenses are sought. | Our permanent footprint of office and laboratory space is adequate for critical business activities after market entry; relevant licenses and permits are in place. |  |
| **Accounting** | **12.Are you aware of accounting needs and practices?** | We do not understand our tax constraints and liabilities; no specialized advisers for our simple accounting methods. | We know a little about tax issues and are seeking outside expertise to help with taxes and accounting. | We have an accounting system in place that recognizes tax liabilities. | We have robust accounting practices and security management. We are exploring tax credit, investment, and depreciation strategies. |  |
| **Governance, Leadership, and Accountability** | **13.What is the governance structure of your business?** | We lack well-defined governance structures. | We are getting business guidance to address immediate objectives. | Our governance structure identifies the responsibilities of key players with resolution procedures for nonperformance and noncompliance. | We have governance systems to monitor the leadership effectiveness. Our governance plan monitors success at managing and achieving our corporate goals. |  |

**Table 3: Design and Production Capabilities**

| **Development Topic** | **Question** | **Maturity Gradient Scale** | | | | **Observations** |
| --- | --- | --- | --- | --- | --- | --- |
|  |  | **Initial** | **Foundational** | **Managed** | **Mature** |  |
| **Facilities/ Utility/ Storage:** | **14.Do your facility/ utility/storage requirements & capabilities match your office & manufacturing needs?** | We have not considered production facility requirements. | Our activities are in a shared/borrowed space with minimal equipment for our immediate needs. OR  We are currently exploring outsourcing vendors. | Our facility and utility requirements are defined for short-term development. OR  We are negotiating with outsourcing companies. | Our facility and utility requirements are appropriate for later stage activities.  Critical utilities are defined.  (In-house or Outsourced) |  |
| **Equipment Readiness**  **(N/A if software only)** | **15.How is your equipment for manufacturing and testing validated and maintained?** | We are working with prototypes and have not considered production equipment. | The available equipment we have is not validated and has little systematic cleaning and maintenance. | Our SOPs for validating and maintaining production equipment are in place and we have completed some testing. | We have systematic validation, calibration and cleaning schedules active for production and testing equipment. |  |
| **Process Flow  (N/A if software only)** | **16a.Do you understand and document processes for producing the product?** | We are in the pre-prototype phase or are working with a “proof of concept” prototype and have minimally considered production needs. | We are currently working prototypes (alpha, beta, etc.) but do not yet have production process flows. | Our production processes are understood but minimally documented. We are exploring design transfer partners and have a pilot production. | Our procedures are documented in reviewed SOPs under a quality management system. Our documentation system is well-organized. |  |
| **Process Flow  (For software only devices)** | **16b. Do you understand and document processes for developing and validating the software?** | We are in the Pre-Development phase and are defining our product vision. | We are in the process of defining our initial set of design and development inputs. | We are working with a vendor to help us develop our software verification and validation processes. | We have an ISO 13485 and FDA QRS compliant software validation procedure in place. |  |
| **Environmental Health and Safety (N/A if software only)** | **17.What are the environmental, health and safety considerations for your production facility?** | We are not yet aware of the requirements for environmental health and safety. OR  Our product does not require these at this stage of development. | We recognize the need to meet safety requirements, but formalized processes do not exist. | We have defined the State and OSHA requirements.  Insurance needs are being explored. | We have addressed the health, safety, and environmental needs in production and most other areas.  We have insurance coverage for fire/other liabilities. |  |
| **Materials: (N/A if software only)** | **18.Do you control the specifications and sourcing of major raw materials/ components/ supplies?** | We are still finalizing the materials that will be used in the product. | We have identified the raw material needs but they are not managed systematically. | We have documented the sourcing of raw materials with vendor files and payment systems in place. | We have a database to document receipt and inspection of materials. The critical quality attributes are defined and procedures for supplier controls are in place. |  |
| **Information Technology (IT)** | **19.What is the state of your IT & network infrastructure?** | We do not have any formal or informal IT structure. | Our IT needs depend on personal computing systems without cybersecurity firewalls or user tracking. The users have equipment, but no systems are defined. | Our IT infrastructure system is defined.  Methods for installing and supporting infrastructure are in place. | Our current and future business needs for IT are defined.  We have equipment and vendors in place for addressing cybersecurity and document control requirements. |  |

**Table 4: Regulatory and Clinical Readiness**

| **Development Topic** | **Question** | **Maturity Gradient Scale** | | | | **Observations** |
| --- | --- | --- | --- | --- | --- | --- |
|  |  | **Initial** | **Foundational** | **Managed** | **Mature** |  |
| **Regulatory Requirements for Development and Registration** | **20. Do you understand how to develop and register medical devices in accordance with US medical device regulations?** | Our knowledge of US medical device regulations is absent or poor. | We understand a little about the US medical device regulations and are hiring a regulatory professional or consultant. | We are aware of product-specific requirements for US marketing approval and have a regulatory professional or consultant. | We address responsibilities throughout the product life cycle. We have a seasoned regulatory staff that monitors FDA interactions and regulatory intelligence. |  |
| **Quality Management System  (N/A if CLASS I exempt device)** | **21. Do you have a Quality Management System (QMS) to formally document processes and procedures to attain high reproducibility?** | We do not understand the requirements for a QMS. | We understand the basic elements of Quality System regulations and are planning to develop a QMS. | We have begun to implement a QMS; we have hired a Quality Assurance manager/ consultant and have begun audits in some areas. | We have a QMS team managing production, risk management and CAPA activities, and have a system for audits and tracking. |  |
| **Pricing and Reimbursement Strategy** | **22. Do you have staff to develop an insurance reimbursement strategy?** | We do not know what is required to develop a reimbursement strategy. | We understand that we must plan for reimbursement but have not engaged a dedicated consultant/ staff member. | We have explored reimbursement strategies and are planning to hire appropriate staff or consultants. | We understand the needs of coverage, coding, and payment as they relate to our product(s). |  |
| **Medical Device Promotional & Advertising (Ad/Promo) Guidelines and Sale Practices** | **23. Are you familiar with permissible promotion and sales practices for medical devices?** | We do not know about regulatory guidelines for promotional and sales practices for medical devices; it is not yet relevant. | We are aware that regulatory constraints affect promotion and sales but have no formal policy. | We have hired consultants to educate and audit us on compliance about promotional and sales practices during clinical trials and commercialization. | Our sales, regulatory affairs and medical affairs staff are trained to review promotional materials; our meeting and sales reps are trained and monitored with respect to HCP interactions. |  |
| **Clinical Evaluation** | **24. Has a clinical focus group evaluated the medical need and design concept for your device?** | We are not familiar with the use of a focus group during development. | We are creating a team of HCPs to evaluate needs and designs. | We have conducted one focus group with HCPs and are incorporating feedback/advice into the device design. | We have conducted more than one focus group with medical professionals and have incorporated their advice/feedback into the design. |  |
| **Clinical Expertise** | **25. Do you have clinical assistance or formal partnerships in the development of the device?** | We do not have any clinical support yet. | We are searching for a clinical partner to conduct any required clinical testing. | We have contracted with at least 1 partner who is able to conduct clinical testing of our device. | We have an established team of partners and a plan for each step of clinical development and testing for our device. |  |

**Table 5: Human Resources and Role Evolution**

| **Development Topic** | **Question** | **Maturity Gradient Scale** | | | | **Observations** |
| --- | --- | --- | --- | --- | --- | --- |
|  |  | **Initial** | **Foundational** | **Managed** | **Mature** |  |
| **HR Governance and Policies** | **26. Do you have Human Relations (HR) structures and policies?** | We do not have any HR policies or employee handbooks. | We understand some HR policies but have not implemented systematic procedures. | Our employee handbook is complete. We are finalizing policies and responsibilities for implementation and enforcement. | We have HR staffing to oversee the implementation and enforcement of HR policies and practices, onboarding, and training. |  |
| **Organization Roles** | **27. Have you defined the roles, titles, and responsibilities of the staff?** | We have no defined roles and are “all hands-on deck.” We have no plan for recruitment or modifications of roles over time. | We are specifying roles for management and development, but everyone reports to the CEO or COO. | We have forecasted our personnel needs for late-stage development and have begun to consider how roles of staff may change as we grow. | Each employee has a job description and responsibilities. An organization chart has a defined reporting structure. We have discussed role evolution and have planned for such change. |  |
| **Legal Compliance** | **28. Are you compliant with federal, state, and local labor laws?** | We do not understand the applicable labor laws for maintaining our business. | We have hired a consultant to teach us about applicable labor laws and to help us design a compliance strategy. | We have a plan in place to conform with applicable labor laws and have a manager to ensure that the plan is followed as part of his/her other job duties. | We have a detailed plan in place. We have hired staff/ consultants to ensure that compliance is maintained and monitored. |  |
| **Payroll and Taxes (N/A if no STAFF)** | **29. Do you have a system to ensure that staff are paid on time and appropriate taxes are withheld?** | We have less than 4 employees who draw little/no salary, are paid through grants or contracts. | All employees are handled like contractors. We are exploring a more formal system with a payroll vendor to assure that we are compliant with payroll and tax laws. | We have a payroll vendor or have hired a staff person to monitor employee time commitments, payments, and relevant tax withholdings. | We have a team to ensure that staff are paid, and taxes are withheld and submitted appropriately. |  |
| **Employee Records (N/A if no STAFF)** | **30. Do you keep employee records, and if so, how do you keep this information confidential?** | We do not keep any records on employment. | We are aware of the statutes and regulations for employee record keeping, but do not have a systematic electronic filing system. | We have a legally compliant record keeping system to ensure disaster and confidentiality protection and protection. We are establishing methods to archive and cull records. | We have purchased a legally compliant electronic data management system (EDMS) for personnel records. We have written procedures for record retention and destruction. |  |

**General SMI feedback questions:**

1. What are your general thoughts about this interview regarding the tool?

2. Specifically, were any questions surprising or not applicable?

3. Do you feel like this exposed you to areas of development you had not considered yet?

4. Would you prefer the format of an interview or a self-survey?
